# Supplementary material for: Improving Drug Discovery by Nucleic Acid Delivery in Engineered Human Microlivers
Source: Cell Metab. 2019 Mar 5;29(3):727–735.e3. doi: 10.1016/j.cmet.2019.02.003 (PMC6408324; doi:10.1016/j.cmet.2019.02.003)
Supplement: Document S1. Figures S1 and S2 and Tables S1 and S2 [file mmc1.pdf]

Cell Metabolism, Volume *29*

Supplemental Information

Improving Drug Discovery by Nucleic Acid Delivery in Engineered Human Microlivers

Liliana Mancio-Silva, Heather E. Fleming, Alex B. Miller, Stuart Milstein, Abigail Liebow, Patrick Haslett, Laura Sepp-Lorenzino, and Sangeeta N. Bhatia

SUPPLEMENTAL DATA

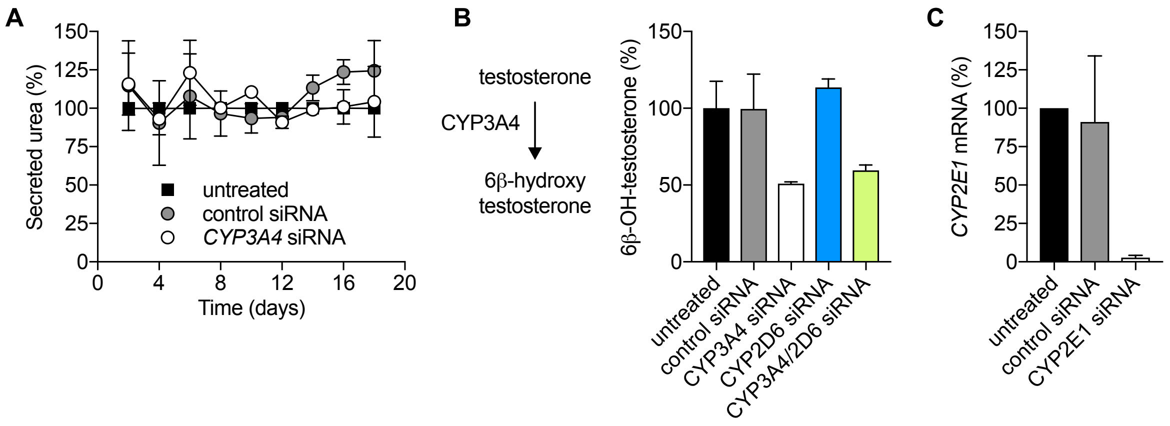

Figure S1. CYP3A4 silencing in MPCCs. **Related to Figures 2 and 3.**
(A) Relative urea levels in the culture supernatant of CYP3A4-silenced MPCCs, as determined by a colorimetric assay. Values are mean ± SEM (n=2). (B) Relative 6β-hydroxytestosterone levels in the supernatant measured by mass spectrometry after incubation with testosterone on day 4 post-transfection (schematic of the reaction shown on the left). 6β-hydroxytestosterone peak area was normalized to untreated cells. Values are mean ± SEM (n=2). (C) *CYP2E1* mRNA levels at day 3 post-transfection. Values (mean ±SEM, n=2) were normalized to untreated MPCCs.

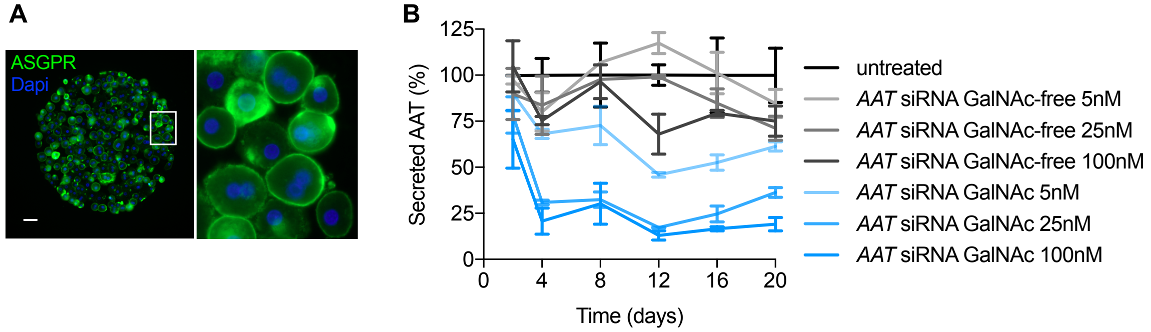

Figure S2. GalNAc-mediated silencing in MPCCs. **Related to Figure 4.**
(A) ASGPR expression in one of the 3 assayed donors at the time of siRNA addition. A representative hepatocyte island is shown, with a zoomed inset on the right. Scale bar, 50 µm. (B) GalNAc- and dose-dependent AAT silencing in free-uptake mode. Values were normalized to untreated cells (mean ± SD). A representative of 3 tested hepatocyte donors is shown.

Table S1. List of GalNAc-conjugated and unconjugated siRNA sequences. **Related to STAR Methods.**

siRNA	Sense (5'-3')	Antisense (3'-5')
CD81	UGCUCUUCGUCUUCAAUUUCA	UGAAAUUGAAGACGAAGAGCAGG
SERPINA1 (AAT)	CACCUGGAAAAUGAACUCACA	UGUGAGUUCAUTUUCCAGGUGCU

Table S2. List of primer sequences. **Related to STAR Methods.**

Primer	Forward	Reverse
CYP3A4	CCGAGTGGATTTCCTTCAGCTG	TGCTCGTGGTTTCATAGCCAGC
CYP2D6	GCAAGAAGTCGCTGGAGCAGTG	CTCACGGCTTTGTCCAAGAGAC
CYP2E1	GAGCACCATCAATCTCTGGACC	CACGGTGATACCGTCCATTGTG
gapdh	GTCTCCTCTGACTTCAACAGCG	ACCACCCTGTTGCTGTAGCCAA
