# Supplementary material for: Improving Drug Discovery by Nucleic Acid Delivery in Engineered Human Microlivers
Source: Cell Metab. 2019 Mar 5;29(3):727–735.e3. doi: 10.1016/j.cmet.2019.02.003 (PMC6408324; doi:10.1016/j.cmet.2019.02.003)
Supplement: Document S2. Article plus Supplemental Information [file mmc2.pdf]

Short Article

Cell Metabolism

Improving Drug Discovery by Nucleic Acid Delivery in Engineered Human Microlivers

Graphical Abstract

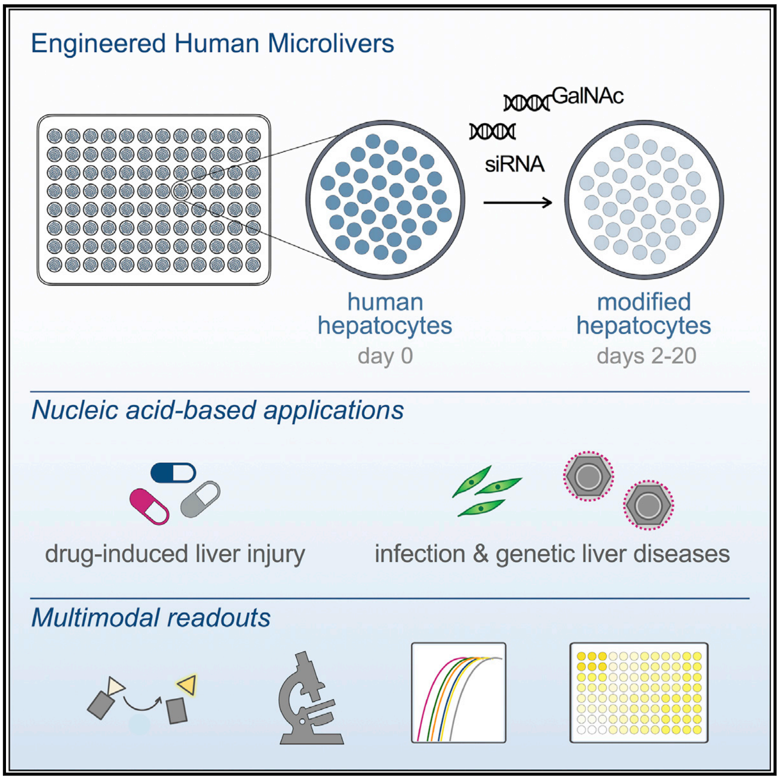

Highlights

- Engineered human microlivers enable robust long-term nucleic acid-mediated silencing
- Human microlivers support assessment of hepatic-targeted nucleic acid-based therapies
- siRNA-mediated silencing effectively modifies hepatocyte drug metabolism

Authors

Liliana Mancio-Silva,
Heather E. Fleming, Alex B. Miller, ...,
Patrick Haslett, Laura Sepp-Lorenzino,
Sangeeta N. Bhatia

Correspondence

sbhatia@mit.edu

In Brief

Mancio-Silva et al. show that nucleic acid-mediated silencing of primary human hepatocytes can be leveraged in an *in vitro* engineered human liver model to fine-tune metabolism and to assess safety and efficacy of RNAi-based therapeutics.

Mancio-Silva et al., 2019, Cell Metabolism *29*, 727–735
March 5, 2019 © 2019 The Authors. Published by Elsevier Inc.
https://doi.org/10.1016/j.cmet.2019.02.003

CellPress

Cell Metabolism
Short Article

Improving Drug Discovery by Nucleic Acid Delivery in Engineered Human Microlivers

Liliana Mancio-Silva,[1] Heather E. Fleming,[1] Alex B. Miller,[1,2] Stuart Milstein,[3] Abigail Liebow,[3] Patrick Haslett,[3] Laura Sepp-Lorenzino,[3,7] and Sangeeta N. Bhatia[1,2,4,5,6,8,*]
[1]Institute for Medical Engineering and Science, Massachusetts Institute of Technology, Cambridge, MA 02142, USA
[2]Broad Institute, Cambridge, MA 02142, USA
[3]Alnylam Pharmaceuticals, 300 3rd Street, Cambridge, MA 02142, USA
[4]Howard Hughes Medical Institute, Chevy Chase, MD 20815, USA
[5]Koch Institute for Integrative Cancer Research, Cambridge, MA 02142, USA
[6]Department of Medicine, Brigham and Women's Hospital, Boston, MA 02115, USA
[7]Present address: Vextex Pharmaceuticals, 50 Northern Avenue, Boston, MA 02210, USA
[8]Lead Contact
*Correspondence: sbhatia@mit.edu
https://doi.org/10.1016/j.cmet.2019.02.003

SUMMARY

The liver plays a central role in metabolism; however, xenobiotic metabolism variations between human hepatocytes and those in model organisms create challenges in establishing functional test beds to detect the potential drug toxicity and efficacy of candidate small molecules. In the emerging areas of RNA interference, viral gene therapy, and genome editing, more robust, long-lasting, and predictive human liver models may accelerate progress. Here, we apply a new modality to a previously established, functionally stable, multi-well bioengineered microliver—fabricated from primary human hepatocytes and supportive stromal cells—in order to advance both small molecule and nucleic acid therapeutic pipelines. Specifically, we achieve robust and durable gene silencing *in vitro* to tune the human metabolism of small molecules, and demonstrate its capacity to query the potential efficacy and/or toxicity of candidate therapeutics. Additionally, we apply this engineered platform to test siRNAs designed to target hepatocytes and impact human liver genetic and infectious diseases.

INTRODUCTION

The liver plays an important role in protecting the organism from various insults, including drugs, chemicals, and pathogens. This protective ability stems from a wide variety of enzymes expressed by hepatocytes that catalyze the oxidation, reduction, and hydrolysis (phase 1) and/or conjugation (phase 2) of functional groups, leading to xenobiotic conversion into metabolites that can be efficiently eliminated from the body. During this detoxification process, some drugs and chemical molecules are converted into highly reactive toxic metabolites that can cause severe cellular damage. Drug-induced liver injury (DILI) remains a leading cause of drug candidate failure in preclinical and clinical trials, as well as a common cause for drugs to be withdrawn from the market after approval (Regev, 2014). In other situations, an otherwise effective parental drug compound can be inactivated by the metabolism process, negating its therapeutic impact, with or without the generation of a toxic by-product (Chen et al., 2018; de Man et al., 2018; Persiani et al., 2006). Thus, accurate prediction of drug metabolism and the mechanism of toxicity in human liver is a critical task to establish an efficient drug discovery pipeline. A major limitation in this area is the lack of *in vitro* or animal models that faithfully recapitulate human hepatic-specific functions. Species differences in drug metabolism, drug targets, and pathophysiology are factors that limit the utility of animals for preclinical assessments (Olson et al., 2000). The alternative *in vitro* liver models include human hepatocarcinoma cell lines and primary hepatocytes, yet these experimental tools also present major challenges. Specifically, hepatocarcinoma cell lines are of limited utility due to uncontrolled proliferation and abnormal hepatic-specific function observed in most cell lines, while primary hepatocytes, which are considered the gold standard to study metabolism and predictive toxicity, are short lived in culture (Soldatow et al., 2013), and sandwich-cultured hepatocytes, which have an extended survival time, have been shown to have altered metabolic function (Jacobsen et al., 2011; Mathijs et al., 2009).

In an effort to overcome the rapid loss of metabolic function observed in cultured primary human hepatocytes, remarkable progress has been made in the bioengineering field to develop technologies that support long-term phenotypic function of *in vitro* cultured primary hepatocytes (Bhatia et al., 2014; Underhill and Khetani, 2017). Engineered liver systems of primary human hepatocytes are available in a variety of platform models, but typically rely on a single hepatocyte donor, which might be problematic due to under sampling the genetic variation seen in phase 1 and phase 2 enzymes across individuals of different genotypes (Kratochwil et al., 2017; Rogue et al., 2012). This population-based heterogeneity has been shown to account for much of the observed clinical variability in drug effectiveness and risk of adverse events (Zanger and Schwab, 2013). An attractive alternative would be the capacity to perform drug

This is an open access article under the CC BY license (http://creativecommons.org/licenses/by/4.0/).

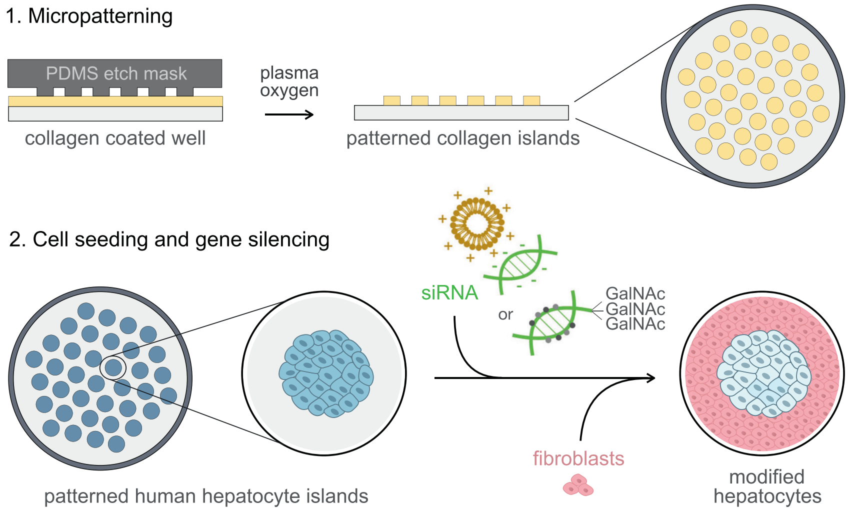

Figure 1. Fabrication of Gene-Silenced MPCCs

Fabrication of MPCCs involves a two-step process: first, patterning of collagen islands in multi-well plates using photolithography techniques, and second, seeding of primary human hepatocytes and stromal supportive fibroblasts to establish the co-culture system. Patterned collagen islands (spots of 500 μm diameter; illustrations represent side and top views of the well) are obtained by applying a silicone elastomer polydimethylsiloxane (PDMS) mask to collagen (yellow)-coated wells, followed by exposure to oxygen plasma. Next, primary human hepatocytes (blue) are seeded and allowed to settle onto the collagen, creating patterned hepatocyte islands (top view). Nucleic acids (siRNA, green) are then included in the media and incubated for 20–24 h, after which mouse fibroblasts (pink) are seeded and allowed to bind in the intervening space between hepatocyte islands. Regular negatively charged siRNAs can be delivered to hepatocytes using cationic lipid transfection reagents, such as lipofectamine (orange). Chemically stabilized siRNAs conjugated to GalNAc are delivered to hepatocytes via interaction with the hepatocyte surface receptor, ASGPR. RNAi-induced gene silencing (light blue) efficiency can be assessed by multiple functional readouts (ELISA for secreted proteins, microscopy or flow cytometry for protein expression, RT-PCR for mRNA transcript abundance, or enzymatic cell assays) and challenged by different experimental perturbations, such as drugs or hepatotropic pathogens (e.g., *Plasmodium* parasites and hepatitis B or C viruses).

screens in higher versus relatively low-metabolizing donors, which could ideally be achieved via genetic engineering of otherwise identical hepatocytes in order to tune potential drug metabolism. Furthermore, accurate prediction of potential toxic responses in a systematic screening platform of this sort would require the use of more metabolically active primary human hepatic cells, rather than transformed cell lines. Our engineered human microlivers have been previously shown to satisfy this latter criterion, in that micropatterned co-cultures (MPCCs) of primary human hepatocytes and supportive stromal cells successfully maintain multiple axes of liver metabolism and function and have been shown to reliably predict the hepatoxicity of FDA-approved and preclinical compounds (Ballard et al., 2016; Davidson et al., 2017; Khetani and Bhatia, 2008; Khetani et al., 2013; Lin and Khetani, 2017; Wang et al., 2010; Ware et al., 2017).

Here, we demonstrate that gene modulation of human hepatocytes can be effectively achieved in a robust, persistent manner in the MPCC system. Specifically, we exploited the endogenous RNA interference (RNAi) pathway to post-transcriptionally silence central drug metabolism genes and assess the impact of these changes on a natural substrate, as well as on DILI assessment of known hepatotoxins. By showing that it is possible to tune drug metabolism by directly manipulating gene expression patterns, we can better model population-wide diversity to screen for potential toxic compounds, or "dial back" key metabolizing pathways that could otherwise mask an effective candidate compound. This new capability can be used to open the door for structure-activity relationship testing of compounds in the setting of both high- and low-metabolizing genotypes. We also demonstrated that novel RNAi-based liver-targeting therapies can be leveraged to model the effectiveness of two emerging alternatives to conventional chemical drugs, one that blocks the production of a toxic secreted protein, AAT, and another that removes a surface molecule required for entry by a hepatotropic pathogen, CD81. Collectively, this study highlights how genetic engineering tools can be applied to fine-tune *in vitro* human liver models to test and develop a wide range of pre-clinical interventions. It also provides a roadmap for the propagation of genetic manipulation of human hepatocytes to other engineered liver systems, such as 3D cultures, liver-on-a-chip, and humanized mouse models.

RESULTS AND DISCUSSION

Engineered Human Microlivers Enable Robust Long-Term Nucleic Acid-Mediated Silencing

An essential part of the drug development process is the analysis of hepatic metabolism of the candidate compound. Hepatic assays can be used to detect, avoid, and/or predict potential human liver toxicity as well as identify drugs with maximal efficacy. However, this practice has been hindered because the available systems do not adequately represent the diversity of human metabolic enzyme expression, nor do these culture systems maintain metabolic function over time. Previously, we have demonstrated that photolithography-based micropatterning and co-culture technologies are valuable tools that maintain primary human hepatocytes with stable hepatic-specific function for weeks (Khetani and Bhatia, 2008) (Figure 1). This platform has been engineered to accommodate 24-, 96-, and 384-well configurations (Gural et al., 2018; March et al., 2013, 2015). MPCCs have been widely used in pre-clinical and post-market testing to assess potential drug toxicity (Khetani et al., 2013; Kratochwil et al., 2017; Ware et al., 2015). More recently, such systems have been used to model hepatotropic infections (Gural et al., 2017, 2018; March et al., 2013; Ng et al., 2014; Ploss et al., 2010; Shlomai et al., 2014), enabling their use in screening for clinical-stage antimalarial drugs and vaccine efficacy (Kato et al., 2016; Kisalu et al., 2018; Phillips et al., 2015). For the purposes of selecting optimally effective compounds, and for predicting DILI, any

screening platform must not only utilize functionally stable human hepatocytes, but also take into account that varied expression of metabolizing enzymes will influence the dose at which a candidate therapeutic might elicit an effective and/or hepatotoxic response. Thus, screening assays must be performed with hepatocytes sourced from a wide range of genotypes, or—preferably, to enable interassay comparisons—assays should incorporate primary hepatocytes with tunable enzyme expression patterns, to reflect the diversity of human metabolism phenotypes. While modulation of metabolic enzymes has been attempted in short-term studies (Chan et al., 2018; Ramsden et al., 2014), it has proven challenging to achieve robust and persistent modification of gene expression in human hepatocytes *in vitro*.

To assess whether MPCC-stabilized human hepatocytes could be successfully targeted for nucleic acid delivery to enable metabolic studies, we probed a major cytochrome P450 (CYP450) phase 1 enzyme, CYP3A4, involved in clinically relevant drug metabolism. Commercially available small interfering RNA (siRNA) oligonucleotide pools specific for *CYP3A4* transcripts were delivered to human hepatocytes via transfection after seeding micropatterned hepatocytes. Scrambled siRNA sequences were added to additional wells as controls. Following overnight exposure to siRNA duplexes, supporting stromal fibroblasts were added to surround the islands of patterned primary hepatocytes, after which the cultures were maintained untreated, other than media changes, for the duration of the experiment (Figure 1). *CYP3A4* silencing efficiency was measured by qRT-PCR and detection of protein by immunofluorescence. Robust reduction of mRNA (Figure 2A) and protein (Figure 2B) was observed in the *CYP3A4* siRNA-treated islands compared to control siRNA or untreated hepatocytes. To assess the impact of the observed protein loss on total enzymatic activity, we utilized a luminescence-based assay that specifically measures cellular CYP3A4 activity by converting a luminogenic substrate into luciferin, which is released into the culture media and further converted into light in the presence of luciferase (Figure 2C). Within 4 days of transfection, a 95% reduction in CYP3A4 activity was observed in the cultures treated with *CYP3A4* siRNAs, relative to the untreated condition. No significant effect was detected in the wells transfected with the control siRNA (Figure 2D). While the degree of knockdown fell just short of 100%, we anticipate that the non-dividing status of the primary hepatocytes will minimize the risk that the residual population of unmodified cells will out-compete those that were successfully modified. To test this hypothesis, we evaluated the durability of the RNAi-induced CYP3A4 silencing by monitoring CYP3A4 enzymatic activity in the same wells over the course of several weeks. CYP3A4 activity levels remained at less than 30% of control samples for at least 20 days post-transfection (Figure 2D). In parallel, we also monitored the levels of secreted hepatocyte-specific biomarkers, such as human albumin, in all the tested conditions. As shown in Figure 2E, exposure to siRNAs (*CYP3A4* or control) did not significantly change albumin production and secretion over the course of the culture period, suggesting that hepatocyte viability was not compromised. Additionally, levels of urea production were similarly unchanged (Figure S1A), and no obvious morphological alterations were observed in the various conditions (data not shown). Loss of CYP3A4 activity after RNAi-mediated silencing was further confirmed by exposure to a natural substrate, testosterone, which is mainly metabolized into 6β-hydroxytestosterone by this enzyme (Figure S1B) (Murayama et al., 2002; Waxman et al., 1988). A short incubation with testosterone was sufficient to differentiate between the function of the *CYP3A4* siRNA treated and controls, with a 50% reduction in 6β-hydroxytestosterone production and secretion (Figure S1B).

Next, we assessed the capability of the co-culture system to support the manipulation of multiple transcripts at the same time. siRNA oligonucleotides against *CYP3A4* and *CYP2D6* were added to micropatterned hepatocytes alone or in combination, and the extent and specificity of induced knockdown were determined by qRT-PCR and enzymatic assays. We observed highly specific and robust 80%–90% reduction of both *CYP3A4* and *CYP2D6* transcripts relative to the untreated or siRNA controls, when the two siRNAs were added simultaneously (Figure 2F). The silencing specificity was further corroborated by functional assays employing exposure to proluciferin (Figure 2G) or natural substrates (Figure S1B).

Nucleic Acid-Mediated Silencing Effectively Modifies Primary Human Hepatocyte Drug Metabolism

Having established that CYP450 activity can be effectively and durably inhibited by siRNA duplexes in primary human hepatocytes, we performed a proof-of-concept toxicity analysis, using known hepatotoxicants, acetaminophen and atorvastatin (Figure 3). Acetaminophen (N-acetyl-p-aminophenol, APAP) is a widely used analgesic that can cause acute hepatic necrosis when administered at high doses, due to the formation of a toxic metabolite (N-acetyl-p-benzoquinone imine, NAPQI) produced when CYP3A4, CYP2E1, CYP1A2, and CYP2A6 act on the parent drug. NAPQI is detoxified by glutathione (GSH) to form an APAP-GSH conjugate, but sufficiently high doses of APAP can lead to 90% depletion of GSH, which results in binding of NAPQI to cellular macromolecules and induction of cell injury (James et al., 2003) (Figure 3A). To evaluate whether siRNA-mediated CYP3A4 or CYP2E1 knockdown in human hepatocytes can blunt APAP toxicity, we assessed albumin secretion in drug-treated cultures with and without siRNAs against *CYP3A4* or *CYP2E1*. Monitoring for reduced albumin production is one readout for hepatocyte damage (Ware et al., 2015). One week after knockdown was initiated, MPCCs were treated with escalating doses of APAP for 24 h. An ~2-fold increase of the half maximal inhibitory concentration (IC_{50}) of APAP was seen in CYP siRNA conditions compared to the control siRNA-treated cells (Figures 3B, 3F, S1C). Thus, by modifying the expression level of CYP3A4 or CYP2E1 enzymes, the toxicity of acute APAP treatment was reduced. To further demonstrate known mechanisms of action, we performed combinatorial experiments using siRNA treatment and a GSH-depleting agent, L-buthionine-sulfoximine (BSO). We observed increased APAP-induced hepatotoxicity in the presence of BSO, which was considerably less pronounced when CYP3A4 activity was silenced (Figures 3C and 3F).

Next, we evaluated the CYP3A4-dependent metabolism and hepatoxicity of atorvastatin. This drug is primarily used as a lipid-depleting agent, owing to its capacity to inhibit the 3-hydroxy-3-methyl-glutaryl-coenzyme A reductase, an enzyme found in liver tissue that plays a key role in production of cholesterol in the body. Atorvastatin is metabolized by CYP3A4 forming

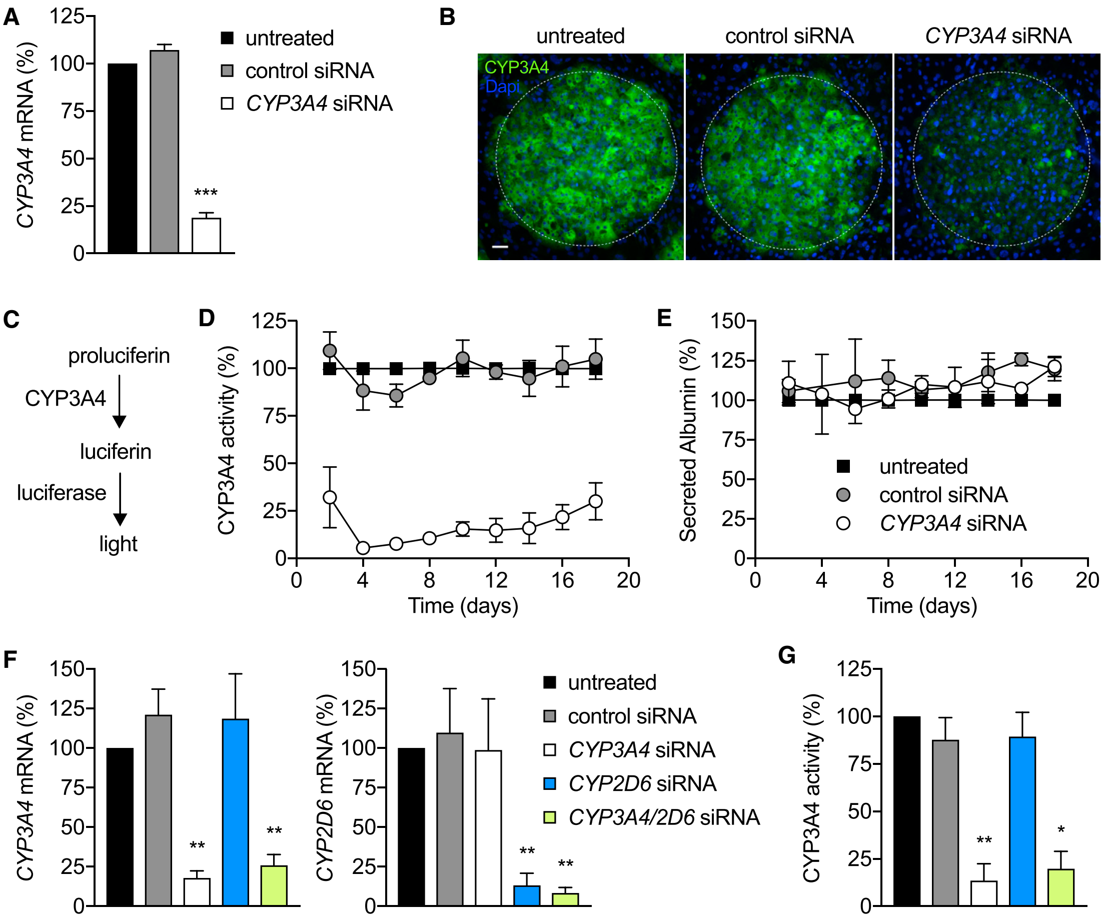

Figure 2. Engineering Liver Metabolism in MPCCs

(A) *CYP3A4* mRNA levels at day 3 post-transfection. Values (mean ± SEM, n = 2) were normalized to untreated MPCCs. $p < 0.001$.

(B) Representative images of hepatocyte islands in MPCCs probed with CYP3A4 antibodies (green) on day 4 post-transfection. Hepatocyte and fibroblast nuclei are shown in blue. The white dotted line delineates the island border of hepatocytes. Scale bar, 50 μm.

(C and D) Schematic reaction of the luminescence-based CYP3A4 activity assay (C) and relative CYP3A4 activity measured in MPCCs (D). Values were normalized to the corresponding untreated cells. Presented data are mean ± SEM (n = 3). $p < 0.0001$.

(E) Relative human albumin levels secreted in MPCCs, as determined by ELISA. Values (mean ± SEM, n = 2) were normalized to the corresponding untreated condition. See Figure S1A for urea levels.

(F) *CYP3A4* and *CYP2D6* mRNA levels at day 3 post-transfection with *CYP3A4* and *CYP2D6* siRNA duplexes, alone or in combination. Graph bars represent mean ± SEM (n = 3). $p < 0.01$.

(G) Relative CYP3A4 activity in the same conditions as (F), using proluciferin. Graph bars represent mean ± SEM (n = 2). **$p < 0.01$. *$p < 0.05$. See Figure S1B for CYP3A4 activity measured with testosterone substrate.

ortho- and parahydroxylated metabolites (Figure 3D), and co-administration of CYP3A4 inhibiting drugs requires close monitoring to avoid increased statin exposure and increased risk of adverse drug reactions, including liver injury (Clarke and Mills, 2006). While the mechanism underlying the atorvastatin-induced hepatotoxicity has not been established, it likely involves increased formation of reactive oxidative species during metabolism (Shu et al., 2016). Using a similar experimental approach to that applied in the APAP studies above, we determined dose-response curves to atorvastatin in human microlivers pre-treated with CYP3A4 or control siRNA. After 24 h incubation, we observed a right-shift of the albumin IC_{50} curve in the CYP3A4 siRNA-treated compared to the control siRNA condition, thus confirming the CYP3A4-dependent mechanism (Figures 3E and 3F).

Our findings corroborate that co-cultured human hepatocytes are able to mediate functional knockdown of essential metabolizing enzymes, but also highlight the capacity to achieve robust and durable *in vitro* modulation of gene expression in these cells. Furthermore, by providing the drug discovery community with an engineered tool that enables the tuning of hepatic metabolism in primary human hepatocytes, this platform addresses an often overlooked and potentially counterintuitive challenge in the field. Namely, screening for new drug candidates in the presence of high-metabolizing hepatocytes can mask a potentially efficacious hit, in that if a toxic metabolite is produced, the otherwise non-toxic parent drug would be discarded. However, if it were possible to test the same drug in a setting of both high- and low-metabolizing hepatic cells, non-toxic parental drugs could be further optimized to bypass the CYP-dependent metabolic

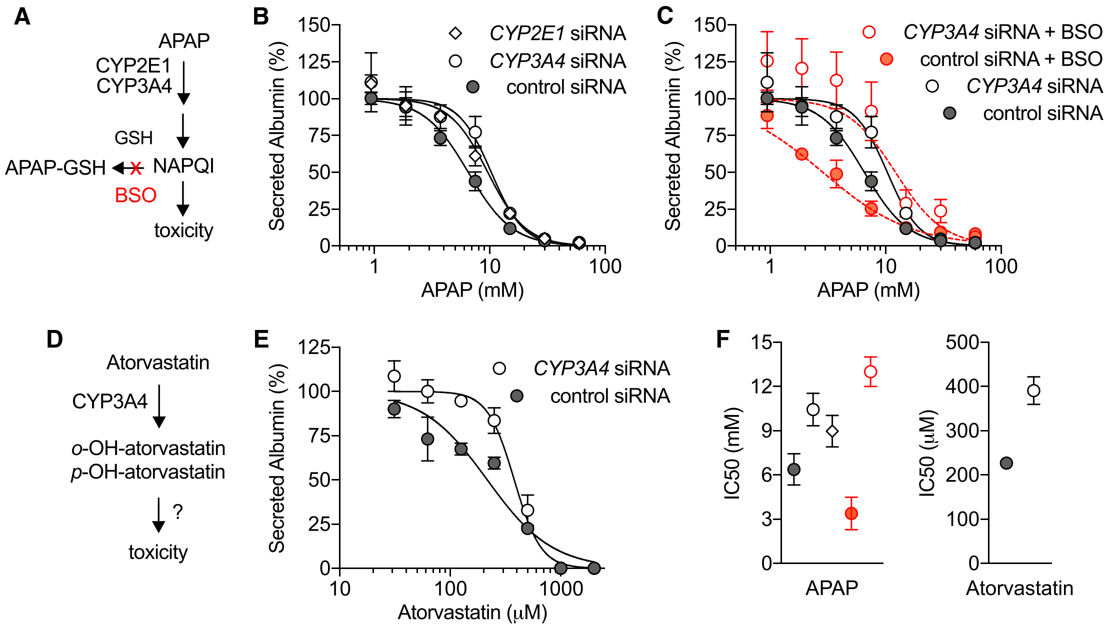

Figure 3. CYP450-Dependent Metabolism and Hepatotoxicity in MPCCs

(A) Schematic representation of APAP metabolism and toxicity.

(B and C) Secreted albumin levels in MPCCs exposed for 24 h to APAP alone (B) or in combination with 200 μM BSO (C) on day 6 post-transfection. Values (mean ± SEM) were normalized to drug-free control wells on each condition (*CYP2E1* siRNA, n = 2; *CYP3A4* siRNA, n = 5; control siRNA, n = 5; control siRNA + BSO, n = 2; *CYP3A4* siRNA + BSO, n = 2). See Figure S1C for *CYP2E1* knockdown efficiency.

(D and E) Schematic of atorvastatin metabolism (D) and albumin levels in MPCCs exposed for 24 h to atorvastatin on day 6 post-transfection (E). Normalized data represent mean ± SEM (n = 2).

(F) Summary of IC_{50} values (mean ± SD) for APAP (left) and atorvastatin (right) in the various conditions. IC_{50} was calculated using non-linear regression variable slope (normalized) analysis.

conversion to an inactive, or even toxic compound. Whereas if the same screen were only conducted in low-metabolizing hepatocytes, the compound could fail in the clinic due to previously undetected DILI. In contrast, if only high-metabolizing cells were utilized, a potentially promising compound may be discarded. Current approaches include utilization of chemical inducers and inhibitors of CYP450 enzymes, but only a few compounds are available and they often lack specificity, thus affecting multiple enzymes. Moreover, these chemicals are only suitable for short-term studies, as they can cause dramatic toxicity. Therefore, the introduction of siRNA-mediated silencing to this *in vitro* system offers a simple and attractive alternative approach to query the mechanistic basis of metabolism and hepatoxicity during drug discovery, and the capacity to mimic patient-specific metabolism profiles without the need for a diverse portfolio of donor cells.

Nucleic Acid Targeting of Hepatocytes as a Candidate Drug Regimen

Nucleic acid therapeutics based on siRNAs are emerging as promising new class of drugs. Thus, we sought to explore whether *in vitro* liver models, such as human MPCC microlivers, might also serve as a screening platform to characterize siRNAs that target the liver. Until recently, nucleic acid delivery has been challenging, but with recent success in targeting siRNAs to the liver there is a need for powerful tools to identify and characterize the clinical utility of siRNAs. Recent reports have highlighted the encouraging clinical potential of targeting siRNA oligonucleotides for use in liver (Huang, 2017). One such approach involves the covalent conjugation of chemically stabilized siRNA oligonucleotides to N-acetylgalactosamine (GalNAc) (Nair et al., 2014; Rajeev et al., 2015), to enable high-affinity binding to the asialoglycoprotein receptor (ASGPR), which is highly expressed on the surface of hepatocytes. Following binding, GalNAc-modified siRNAs are taken up into the cytosol via clathrin-mediated endocytosis and are loaded into the RNA-induced silencing complex (RISC). As a first step in evaluating whether these modified siRNAs would be well-suited for screening using our engineered *in vitro* microlivers, we determined the expression levels of ASGPR, based on immunofluorescence staining of non-permeabilized hepatocytes, assayed at the time of siRNA addition (Figure S2A). This assay represents an important step when using primary human hepatocyte-based microlivers for receptor-mediated interventions, since we have previously seen that not all human hepatocyte sources express equivalent protein levels and/or are similarly susceptible to receptor-mediated processes, such as infection (March et al., 2013; Shlomai et al., 2014). Next, we assayed a set of GalNAc-conjugated and unconjugated siRNA molecules for their capacity to mediate gene silencing in MPCCs by free uptake. We tested siRNA duplexes specific to alpha-1 antitrypsin (*AAT*) (Ascha et al., 2015). AAT-associated liver disease is observed in patients expressing a mutant AAT allele that results in AAT protein misfolding, aggregation, and accumulation. By knocking down AAT in patients

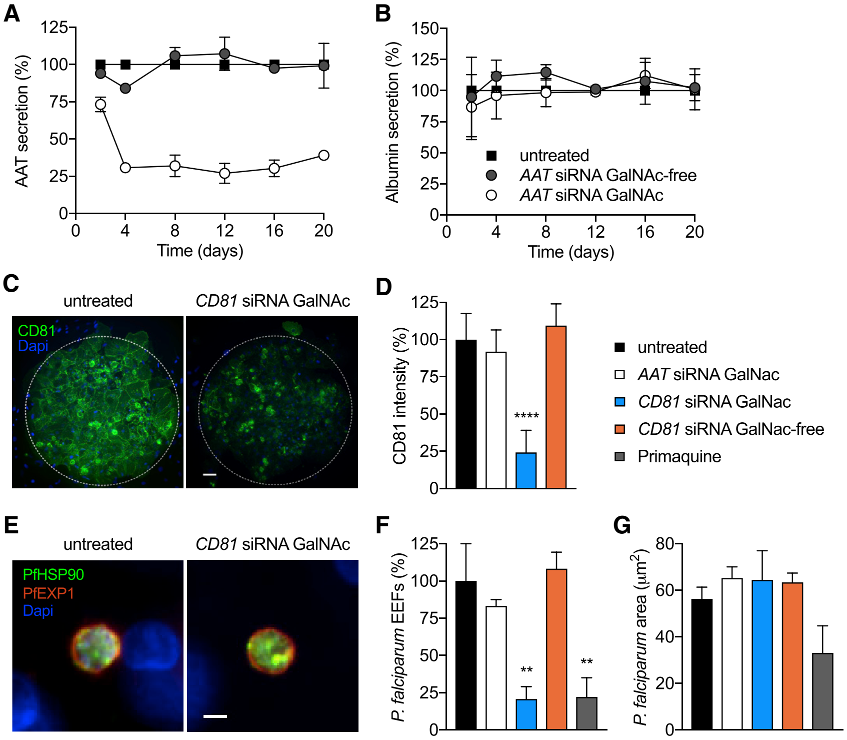

Figure 4. GalNAc siRNA Conjugates Elicit Functional Intervention in MPCCs

(A and B) Secreted AAT (A) and albumin (B) levels determined by ELISA in supernatants of MPCCs treated with 25 nM of GalNAc-conjugated or GalNAc-free *AAT* siRNA, in free-uptake mode. Values were normalized to untreated cells. Data represent the mean ± SEM (n = 3); each independent experiment was performed with a different hepatocyte donor. See Figure S2B for dose-dependent silencing over time.

(C and D) CD81 hepatocyte surface expression in MPCCs 3 days post-treatment with *CD81* siRNA GalNAc-conjugated or GalNAc-free control siRNA, or *AAT* (off-target) GalNAc-conjugated siRNA (25 nM). Representative images of hepatocyte islands immunostained with anti-CD81 antibodies (green) are shown in (C). Nuclei of hepatocytes and fibroblasts are shown in blue. The white dotted line depicts the border of the island of hepatocytes. Scale bar, 50 μm. (D) Graph bars represent mean ± SD of CD81 fluorescence intensity staining per island. $p < 0.0001$.

(E–G) *P. falciparum* infection in MPCCs treated with 25 nM siRNA 3 days prior to infection. Primaquine treatment (250 nM) was initiated 3 h post-infection and repeated daily. MPCCs were fixed at 4 days post-infection and analyzed by immunofluorescence assays.

(E) Representative images of *P. falciparum* hepatic forms. Parasites were detected using antibodies against the parasitophorous vacuolar membrane *P. falciparum* exported protein 1 (PfEXP1, red) and the cytoplasmic heat shock protein 90 (PfHSP90, green). Scale bar, 5 μm.

(F) Quantification of parasite numbers (EEFs, exoerythrocytic forms) per well (mean ± SEM). $p < 0.01$.

(G) Quantification parasite size distribution (mean ± SD). Data represent one of two independent infections.

with the genetic defect, their liver burden could be reduced significantly. We observed a robust dose- and GalNAc-dependent decrease in secreted AAT (Figures 4A and S2B) from normal, primary human hepatocytes, suggesting efficient uptake of the modified *AAT* siRNA molecules in the absence of transfection reagent. Similar to what has been reported in preclinical and clinical trials, we observed that GalNAc siRNA-mediated silencing is sustained for several weeks in our *in vitro* system (Figure 4A). Consistent hepatocyte viability was observed when using secreted albumin as a proxy for hepatocyte health (Figure 4B). Collectively, these findings highlight that it is possible to leverage engineered human microlivers to test the efficacy, and potentially also the risk of toxicity, of clinically relevant GalNAc-decorated siRNA oligonucleotides. From a translational perspective, the availability of such a validated screening tool to use *in vitro*, prior to advancing to clinical development, is an unquestionable alternative to animal models that are currently employed to down-select hepatotoxic GalNAc-conjugated siRNAs (Janas et al., 2018).

Leveraging Nucleic Acid-Mediated Silencing to Intervene in Hepatotropic Infections

The liver is targeted by numerous infectious pathogens that use the hepatocyte as an entry point and site of massive replication in human hosts. To explore the potential therapeutic application of GalNAc siRNA conjugates in preventing or treating hepatotropic infections, we turned to CD81, a tetraspanin integral membrane protein expressed by hepatocytes and required for malaria-causing parasites *Plasmodium falciparum* to enter into their human host (Manzoni et al., 2017; Silvie et al., 2003). First, we confirmed by immunofluorescence staining of non-permeabilized cells that *CD81* siRNA-GalNAc conjugates added to patterned human hepatocytes in free uptake mode resulted in robust depletion of CD81 protein on the surface of hepatocytes, relative to the GalNAc-free *CD81* siRNA, GalNAc-conjugated *AAT* siRNA, or untreated control wells (Figures 4C and 4D). Next, we infected the pre-treated hepatocytes with *P. falciparum* parasites freshly isolated from infected mosquitoes. In parallel, we treated control wells with a known liver-acting antimalarial drug, primaquine. In previous work, we have demonstrated that MPCCs enable full 7-day liver-stage development of *P. falciparum* parasites, leading to replication and formation of new infectious parasites, which can then infect human erythrocytes, mimicking a natural infection (March et al., 2013, 2015; Ng et al., 2014). To determine the impact of GalNAc-conjugated *CD81* siRNA treatment, we employed fluorescence microscopy and used parasite-specific markers to quantify the number of infected hepatocytes and measure the area occupied by intracellular parasite, as a proxy for parasite development, at 4 days post-infection. We observed an 80% reduction in the number of parasites present in wells treated with GalNAc-conjugated *CD81* siRNA, similar to the impact of primaquine treatment (Figures 4E–4G), though the few parasites that were detected within the cultures did not exhibit a reduction in their

development. Unconjugated *CD81* siRNA or siRNA GalNAc-conjugates against an irrelevant sequence (*AAT*) elicited no significant change in the infection by malaria parasites relative to the untreated controls (Figure 4F). Collectively, these results support the relevance of applying engineered liver models, such as MPCC, as screening and discovery tools to test the utility of GalNAc-conjugated siRNA molecules for the treatment or prophylaxis of liver diseases.

Conclusion

The complexity of human physiology has historically made it challenging to design new drugs that are efficacious yet induce minimal toxicity. The biology of the human liver has made the drug discovery process even more challenging, given the multitude of essential functions this organ serves, and the species-specific and context-specific behavior of its hepatocytes. A wide variety of engineering tools have been brought to bear in the effort to establish culture conditions that make human hepatocyte studies amenable to scaled-up screening, while still maintaining their essential functional output. In this study, we explored the capacity to manipulate gene expression in primary human hepatocytes by employing various RNAi-based agents and delivery systems. We demonstrated robust and durable gene silencing of essential hepatic metabolizing enzymes, intracellular pathological proteins, and surface molecules that permit infection by liver pathogens using a 2D-engineered micropatterned co-culture system. This work validated RNAi as an approach not only to study primary human hepatocytes, but also to assess hepatic-targeted nucleic acid agents, and achieve direct modification of drug metabolism. In so doing, we envision a path to enable more accurate and efficient identification of promising chemical compounds and small molecule drugs by detecting potential toxic reactions elicited by both parent and metabolized drug products, as well as the capacity to mimic patient-specific metabolism profiles without the need for a diverse portfolio of donor cells. Finally, we believe the impact of this study will extend beyond RNAi to other nucleic acid tools and therapies (e.g., adeno-associated viral delivery) for which there are not currently adequate human model systems, and expect that these collective genetic tools could likewise be extended to other engineered liver models in the future.

Limitations of Study

We acknowledge that the work presented here only addresses the use of a single form of nucleic acids, siRNA, and also that we have thus far only examined reduction in gene expression, rather than presenting efforts to overexpress a gene of interest. Furthermore, the reduction we observe does not achieve full knockdown, and thus if either complete deletion of a gene were desired or if a protein with a faster turnover rate were under investigation, an alternate approach to gene modification may be more appropriate. Finally, in the experiments described above, the highest efficiency was obtained when silencing of human hepatocyte gene expression was induced prior to the formation of the co-culture platform, i.e., when siRNA agents were added to hepatocyte-only cultures. Thus, kinetic experiments designed to query the impact of silencing in established MPCC cultures would not be supported by the standard platform, without additional modifications that we are currently exploring.

STAR★METHODS

Detailed methods are provided in the online version of this paper and include the following:

- KEY RESOURCES TABLE
- CONTACT FOR REAGENT AND RESOURCE SHARING
- EXPERIMENTAL MODEL AND SUBJECT DETAILS
  - Cells
  - Parasites
- METHOD DETAILS
  - Co-culture and siRNA Treatment
  - RNA Extraction and RT-PCR
  - Immunofluorescence Assay
  - Biochemical Assays
  - *Plasmodium* Infection and Quantification
- QUANTIFICATION AND STATISTICAL ANALYSIS

SUPPLEMENTAL INFORMATION

Supplemental Information can be found with this article online at https://doi.org/10.1016/j.cmet.2019.02.003.

ACKNOWLEDGMENTS

The authors would like to thank Salman Khetani and Vyas Ramanan for discussions and advice, and Sandra March, Nil Gural, and Niketa Nerurkar for technical assistance. This work was supported in part by the Bill & Melinda Gates Foundation (OPP1023607), Alnylam Pharmaceuticals, and the Koch Institute Support Grant P30-CA14051 from the National Cancer Institute. S.N.B. is an HHMI Investigator.

AUTHOR CONTRIBUTIONS

L.M.-S., H.E.F., and S.N.B. conceived the study; L.M.-S. and A.B.M. performed the experiments and analyzed the data; and S.M., A.L., P.H., and L.S.-L. provided reagents and expertise. L.M.-S. and H.E.F. prepared the manuscript with input from all authors.

DECLARATION OF INTERESTS

S.M., A.L., and P.H. are employees of Alnylam Pharmaceuticals. L.S.-L. has moved from Alnylam Pharmaceuticals to Vertex Pharmaceuticals during the preparation of this manuscript. S.N.B. is a director at Vertex Pharmaceuticals; co-founder and consultant at Glympse Bio; consultant for Cristal, Maverick, Synlogic, and Moderna; and receives sponsored research funds from Johnson & Johnson and Alnylam Pharmaceuticals. S.N.B. is an inventor on a patent (WO2006127768A2) related to this research.

Received: July 31, 2018
Revised: November 21, 2018
Accepted: February 11, 2019
Published: March 5, 2019

REFERENCES

Ascha, M., Ascha, M., Dweik, R.A., Zein, N.N., Brown, J.M., Hazen, S.L., Grove, D., and Hanouneh, I.A. (2015). 279 pre-clinical evaluation of ALN-AAT to ameliorate liver disease associated with alpha-1-antitrypsin deficiency. Gastroenterology *148*, S-975.

Ballard, T.E., Wang, S., Cox, L.M., Moen, M.A., Krzyzewski, S., Ukairo, O., and Obach, R.S. (2016). Application of a micropatterned cocultured hepatocyte

system to predict preclinical and human-specific drug metabolism. Drug Metab. Dispos. *44*, 172–179.

Bhatia, S.N., Underhill, G.H., Zaret, K.S., and Fox, I.J. (2014). Cell and tissue engineering for liver disease. Sci. Transl. Med. *6*, 245sr2.

Chan, C.Y.S., Roberts, O., Rajoli, R.K.R., Liptrott, N.J., Siccardi, M., Almond, L., and Owen, A. (2018). Derivation of CYP3A4 and CYP2B6 degradation rate constants in primary human hepatocytes: a siRNA-silencing-based approach. Drug Metab. Pharmacokinet. *33*, 179–187.

Chen, G., Højer, A.M., Areberg, J., and Nomikos, G. (2018). Vortioxetine: clinical pharmacokinetics and drug interactions. Clin. Pharmacokinet. *57*, 673–686.

Clarke, A.T., and Mills, P.R. (2006). Atorvastatin associated liver disease. Dig. Liver Dis. *38*, 772–777.

Davidson, M.D., Kukla, D.A., and Khetani, S.R. (2017). Microengineered cultures containing human hepatic stellate cells and hepatocytes for drug development. Integr. Biol. *9*, 662–677.

de Man, F.M., Goey, A.K.L., van Schaik, R.H.N., Mathijssen, R.H.J., and Bins, S. (2018). Individualization of irinotecan treatment: a review of pharmacokinetics, pharmacodynamics, and pharmacogenetics. Clin. Pharmacokinet. *57*, 1229–1254.

Gural, N., Mancio-Silva, L., He, J., and Bhatia, S.N. (2017). Engineered livers for infectious diseases. Cell Mol. Gastroenterol. Hepatol. *5*, 131–144.

Gural, N., Mancio-Silva, L., Miller, A.B., Galstian, A., Butty, V.L., Levine, S.S., Patrapuvich, R., Desai, S.P., Mikolajczak, S.A., Kappe, S.H.I., et al. (2018). in vitro culture, drug sensitivity and transcriptome of Plasmodium vivax hypnozoites. Cell Host Microbe *23*, 395–406.e4.

Huang, Y. (2017). Preclinical and clinical advances of GalNAc-decorated nucleic acid therapeutics. Mol. Ther. Nucleic Acids *6*, 116–132.

Jacobsen, J.K., Jensen, B., Skonberg, C., Hansen, S.H., and Badolo, L. (2011). Time-course activities of Oct1, Mrp3, and cytochrome P450s in cultures of cryopreserved rat hepatocytes. Eur. J. Pharm. Sci. *44*, 427–436.

James, L.P., Mayeux, P.R., and Hinson, J.A. (2003). Acetaminophen-induced hepatotoxicity. Drug Metab. Dispos. *31*, 1499–1506.

Janas, M.M., Schlegel, M.K., Harbison, C.E., Yilmaz, V.O., Jiang, Y., Parmar, R., Zlatev, I., Castoreno, A., Xu, H., Shulga-Morskaya, S., et al. (2018). Selection of GalNAc-conjugated siRNAs with limited off-target-driven rat hepatotoxicity. Nat. Commun. *9*, 723.

Kato, N., Comer, E., Sakata-Kato, T., Sharma, A., Sharma, M., Maetani, M., Bastien, J., Brancucci, N.M., Bittker, J.A., Corey, V., et al. (2016). Diversity-oriented synthesis yields novel multistage antimalarial inhibitors. Nature *538*, 344–349.

Khetani, S.R., and Bhatia, S.N. (2008). Microscale culture of human liver cells for drug development. Nat. Biotechnol. *26*, 120–126.

Khetani, S.R., Kanchagar, C., Ukairo, O., Krzyzewski, S., Moore, A., Shi, J., Aoyama, S., Aleo, M., and Will, Y. (2013). Use of micropatterned cocultures to detect compounds that cause drug-induced liver injury in humans. Toxicol. Sci. *132*, 107–117.

Kisalu, N.K., Idris, A.H., Weidle, C., Flores-Garcia, Y., Flynn, B.J., Sack, B.K., Murphy, S., Schön, A., Freire, E., Francica, J.R., et al. (2018). A human monoclonal antibody prevents malaria infection by targeting a new site of vulnerability on the parasite. Nat. Med. *24*, 408–416.

Kratochwil, N.A., Meille, C., Fowler, S., Klammers, F., Ekiciler, A., Molitor, B., Simon, S., Walter, I., McGinnis, C., Walther, J., et al. (2017). Metabolic profiling of human long-term liver models and hepatic clearance predictions from in vitro data using nonlinear mixed-effects modeling. AAPS J. *19*, 534–550.

Lin, C., and Khetani, S.R. (2017). Micropatterned co-cultures of human hepatocytes and stromal cells for the assessment of drug clearance and drug-drug interactions. Curr. Protoc. Toxicol. *72*, 1–23.

Manzoni, G., Marinach, C., Topçu, S., Briquet, S., Grand, M., Tolle, M., Gransagne, M., Lescar, J., Andolina, C., Franetich, J.-F., et al. (2017). *Plasmodium* P36 determines host cell receptor usage during sporozoite invasion. eLife *6*, https://doi.org/10.7554/eLife.25903.

March, S., Ng, S., Velmurugan, S., Galstian, A., Shan, J., Logan, D.J., Carpenter, A.E., Thomas, D., Sim, B.K.L., Mota, M.M., et al. (2013). A microscale human liver platform that supports the hepatic stages of Plasmodium falciparum and vivax. Cell Host Microbe *14*, 104–115.

March, S., Ramanan, V., Trehan, K., Ng, S., Galstian, A., Gural, N., Scull, M.A., Shlomai, A., Mota, M.M., Fleming, H.E., et al. (2015). Micropatterned coculture of primary human hepatocytes and supportive cells for the study of hepatotropic pathogens. Nat. Protoc. *10*, 2027–2053.

Mathijs, K., Kienhuis, A.S., Brauers, K.J.J., Jennen, D.G.J., Lahoz, A., Kleinjans, J.C.S., and van Delft, J.H.M. (2009). Assessing the metabolic competence of sandwich-cultured mouse primary hepatocytes. Drug Metab. Dispos. *37*, 1305–1311.

Murayama, N., Nakamura, T., Saeki, M., Soyama, A., Saito, Y., Sai, K., Ishida, S., Nakajima, O., Itoda, M., Ohno, Y., et al. (2002). CYP3A4 gene polymorphisms influence testosterone 6beta-hydroxylation. Drug Metab. Pharmacokinet *17*, 150–156.

Nair, J.K., Willoughby, J.L., Chan, A., Charisse, K., Alam, M.R., Wang, Q., Hoekstra, M., Kandasamy, P., Kel'in, A.V., Milstein, S., et al. (2014). Multivalent N-acetylgalactosamine-conjugated siRNA localizes in hepatocytes and elicits robust RNAi-mediated gene silencing. J. Am. Chem. Soc. *136*, 16958–16961.

Ng, S., March, S., Galstian, A., Hanson, K., Carvalho, T., Mota, M.M., and Bhatia, S.N. (2014). Hypoxia promotes liver-stage malaria infection in primary human hepatocytes in vitro. Dis. Model. Mech. *7*, 215–224.

Olson, H., Betton, G., Robinson, D., Thomas, K., Monro, A., Kolaja, G., Lilly, P., Sanders, J., Sipes, G., Bracken, W., et al. (2000). Concordance of the toxicity of pharmaceuticals in humans and in animals. Regul. Toxicol. Pharmacol *32*, 56–67.

Persiani, S., D'Amato, M., Jakate, A., Roy, P., Wangsa, J., Kapil, R., and Rovati, L.C. (2006). Pharmacokinetic profile of dexloxiglumide. Clin. Pharmacokinet. *45*, 1177–1188.

Phillips, M.A., Lotharius, J., Marsh, K., White, J., Dayan, A., White, K.L., Njoroge, J.W., El Mazouni, F., Lao, Y., Kokkonda, S., et al. (2015). A long-duration dihydroorotate dehydrogenase inhibitor (DSM265) for prevention and treatment of malaria. Sci. Transl. Med. *7*, 296ra111.

Ploss, A., Khetani, S.R., Jones, C.T., Syder, A.J., Trehan, K., Gaysinskaya, V.A., Mu, K., Ritola, K., Rice, C.M., and Bhatia, S.N. (2010). Persistent hepatitis C virus infection in microscale primary human hepatocyte cultures. Proc. Natl. Acad. Sci. U S A *107*, 3141–3145.

Rajeev, K.G., Nair, J.K., Jayaraman, M., Charisse, K., Taneja, N., O'Shea, J., Willoughby, J.L., Yucius, K., Nguyen, T., Shulga-Morskaya, S., et al. (2015). Hepatocyte-specific delivery of siRNAs conjugated to novel non-nucleosidic trivalent N-acetylgalactosamine elicits robust gene silencing in vivo. ChemBioChem *16*, 903–908.

Ramsden, D., Tweedie, D.J., Chan, T.S., and Tracy, T.S. (2014). Altered CYP2C9 activity following modulation of CYP3A4 levels in human hepatocytes: an example of protein-protein interactions. Drug Metab. Dispos. *42*, 1940–1946.

Regev, A. (2014). Drug-induced liver injury and drug development: industry perspective. Semin. Liver Dis. *34*, 227–239.

Rogue, A., Lambert, C., Spire, C., Claude, N., and Guillouzo, A. (2012). Interindividual variability in gene expression profiles in human hepatocytes and comparison with HepaRG cells. Drug Metab. Dispos. *40*, 151–158.

Shlomai, A., Schwartz, R.E., Ramanan, V., Bhatta, A., de Jong, Y.P., Bhatia, S.N., and Rice, C.M. (2014). Modeling host interactions with hepatitis B virus using primary and induced pluripotent stem cell-derived hepatocellular systems. Proc. Natl. Acad. Sci. U S A *111*, 12193–12198.

Shu, N., Hu, M., Ling, Z., Liu, P., Wang, F., Xu, P., Zhong, Z., Sun, B., Zhang, M., Li, F., et al. (2016). The enhanced atorvastatin hepatotoxicity in diabetic rats was partly attributed to the upregulated hepatic Cyp3a and SLCO1B1. Sci. Rep. *6*, 33072.

Silvie, O., Rubinstein, E., Franetich, J.-F., Prenant, M., Belnoue, E., Rénia, L., Hannoun, L., Eling, W., Levy, S., Boucheix, C., and Mazier, D. (2003). Hepatocyte CD81 is required for Plasmodium falciparum and Plasmodium yoelii sporozoite infectivity. Nat. Med. *9*, 93–96.

Soldatow, V.Y., Lecluyse, E.L., Griffith, L.G., and Rusyn, I. (2013). *In vitro* models for liver toxicity testing. Toxicol. Res. (Camb.) *2*, 23–39.

Underhill, G.H., and Khetani, S.R. (2017). Bioengineered liver models for drug testing and cell differentiation studies. Cell Mol. Gastroenterol. Hepatol. *5*, 426–439.e1.

Wang, W.W., Khetani, S.R., Krzyzewski, S., Duignan, D.B., and Obach, R.S. (2010). Assessment of a micropatterned hepatocyte coculture system to generate major human excretory and circulating drug metabolites. Drug Metab. Dispos. *38*, 1900–1905.

Ware, B.R., Berger, D.R., and Khetani, S.R. (2015). Prediction of drug-induced liver injury in micropatterned co-cultures containing iPSC-derived human hepatocytes. Toxicol. Sci. *145*, 252–262.

Ware, B.R., McVay, M., Sunada, W.Y., and Khetani, S.R. (2017). Exploring chronic drug effects on microengineered human liver cultures using global gene expression profiling. Toxicol. Sci. *157*, 387–398.

Waxman, D.J., Attisano, C., Guengerich, F.P., and Lapenson, D.P. (1988). Human liver microsomal steroid metabolism: identification of the major microsomal steroid hormone 6 beta-hydroxylase cytochrome P-450 enzyme. Arch. Biochem. Biophys. *263*, 424–436.

Zanger, U.M., and Schwab, M. (2013). Cytochrome P450 enzymes in drug metabolism: regulation of gene expression, enzyme activities, and impact of genetic variation. Pharmacol. Ther. *138*, 103–141.

STAR★METHODS

KEY RESOURCES TABLE

REAGENT or RESOURCE	SOURCE	IDENTIFIER
Antibodies		
CYP3A4 mouse monoclonal	Thermo Fisher Scientific	Cat# MA5-17064; RRID: AB_2538535
CD81 mouse monoclonal	Santa Cruz Biotechnology	Cat# sc-23962; RRID: AB_627192
ASGPR rabbit polyclonal	Abcam	Cat# ab88042; RRID: AB_10670301
Alexa 635-conjugated anti-mouse	Thermo Fisher Scientific	Cat# A-31575; RRID: AB_2536185
Alexa 594-conjugated anti-rabbit	Thermo Fisher Scientific	Cat# A-11035; RRID: AB_143051
Goat anti-Human Albumin	Bethyl	Cat# A80-129; RRID: AB_67016
Goat anti-Human Albumin HRP Conjugated	Bethyl	Cat# A80-129AP; RRID: AB_67019
Goat anti-Human Alpha-1-Antitrypsin	Bethyl	Cat# A80-122; RRID: AB_67027
Goat anti-Human Alpha-1-Antitrypsin HRP Conjugated	Bethyl	Cat# A80-122P; RRID: AB_2285693
PfHsp90 rabbit polyclonal	StressMarq Biosciences	Cat# SPC-187
PfHsp70 mouse monoclonal (clone 4C9)	gift of Fidel Zavala (John Hopkins University)	N/A
PfEXP1 mouse monoclonal (clone 5.1)	The European Malaria Reagent Repository	http://www.malariaresearch.eu/
Biological Samples		
Primary human hepatocytes	BioIVT	Cat# M00995, F00995
Chemicals, Peptides, and Recombinant Proteins		
Testosterone	Sigma-Aldrich	Cat# T5411
Acetaminophen	Sigma-Aldrich	Cat# A7085
L-Buthionine-(S,R)-Sulfoximine	Cayman chemical	Cat# 14484
Atorvastatin	Cayman chemical	Cat# 10493
Critical Commercial Assays		
P450-Glo CYP3A4 Assay	Promega	Cat# V9002
Urea Nitrogen Test	Stanbio	Cat# 0580250
Experimental Models: Cell Lines		
J2-3T3 murine embryonic fibroblasts	gift of Howard Green (Harvard Medical School)	N/A
Experimental Models: Organisms/Strains		
Plasmodium falciparum NF54	John Hopkins University and Sanaria Inc.	N/A
Oligonucleotides		
ON-TARGETplus CYP3A4 siRNA	Dharmacon	Cat# L-008169010005
ON-TARGETplus CYP2D6 siRNA	Dharmacon	Cat# L-009550-02-0005
ON-TARGETplus CYP2E1 siRNA	Dharmacon	Cat# L-010134-01-0005
ON-TARGETplus Non-targeting Control Pool	Dharmacon	Cat# D-001810-10-05
GalNAc siRNA sequences in Table S1	Alnylam Pharmaceuticals	N/A
Primer sequences in Table S2	Integrated DNA Technologies	N/A
Software and Algorithms		
Image Processing and Analysis in Java	ImageJ	https://imagej.nih.gov/ij/
NIS-Elements Advanced Research	Nikon Instruments	https://www.microscope.healthcare.nikon.com/products/software/nis-elements/nis-elements-advanced-research
GraphPad Prism 7	GraphPad	https://www.graphpad.com/scientific-software/prism/

CONTACT FOR REAGENT AND RESOURCE SHARING

Further information and requests for resources and reagents should be directed to and will be fulfilled by the Lead Contact, Sangeeta N. Bhatia (sbhatia@mit.edu).

EXPERIMENTAL MODEL AND SUBJECT DETAILS

Cells

Cryopreserved primary human hepatocytes were purchased from BioIVT, a vendor permitted to sell products derived from human organs procured in the United States of America by federally designated Organ Procurement Organizations. Donors included a male aged 48 (donor 1), a female aged 35 (donor 2), and male aged 30 (donor 3). Experiments in Figures 2, 3, 4D–4F, and S1 were performed using hepatocytes originated from donor 1. Experiments in Figures 4A, 4B, and S2 comprised donors 1, 2 and 3. Human hepatocytes were maintained in Dulbecco's Modified Eagle Medium (DMEM, Corning) with 10% fetal bovine serum (FBS, GIBCO), 1% ITS (insulin/transferrin/selenous acid and linoleic acid, BD Biosciences), 7 ng/mL glucagon (Sigma-Aldrich), 40 ng/mL dexamethasone (Sigma-Aldrich), 15 mM HEPES (GIBCO), and 100 μg/mL penicillin-streptomycin (Corning). Cells were kept at 37°C in a 5% CO2 environment.

J2-3T3 male murine embryonic fibroblasts (gift of Howard Green, Harvard Medical School) were cultured at < 20 passages in medium comprising of DMEM, 10% (v/v) bovine serum (Thermo Fisher Scientific), and 100 μg/mL penicillin-streptomycin (Corning), and were kept at 37°C in a 5% CO2 environment.

Parasites

P. falciparum sporozoites were isolated via dissection of salivary glands of *Anopheles stephensi* female mosquitoes bred and infected at John Hopkins University Malaria Research Institute (Baltimore, MD, USA) or Sanaria (Rockville, MD, USA). Dissection media comprised DMEM supplemented with 300 μg/mL penicillin-streptomycin (Corning).

METHOD DETAILS

Co-culture and siRNA Treatment

Primary human hepatocytes were seeded on collagen-coated micropatterned 96-well plates as detailed previously (March et al., 2015). siRNA oligonucleotides were added to patterned hepatocytes after washing off the unbound hepatocytes. ON-TARGETplus SMARTpool siRNA oligonucleotides (Dharmacon) were delivered to hepatocytes by using RNAiMAX Transfection Reagent (Thermo Fisher Scientific) per manufacture's protocols at final concentration 100nM in antibiotic-free DMEM media supplemented with 10% FBS (final volume 100 μL). GalNAc siRNA conjugates (Alnylam Pharmaceuticals; Table S1) were added to hepatocytes in a free-uptake mode, in various concentrations in supplemented hepatocyte media. Duplicate or triplicate wells-containing hepatocytes were exposed to siRNA duplexes overnight for 20-24 hr, and subsequently surrounded with 3T3-J2 fibroblasts and cultured in supplemented hepatocyte media.

RNA Extraction and RT-PCR

MPCCs were lysed and homogenized in TRIzol (Thermo Fisher Scientific) after media removal. Total RNA was isolated via chloroform extraction and purified using the RNeasy MinElute Cleanup Kit (QIAGEN). cDNA synthesis was performed using SuperScript II (Thermo Fisher Scientific) and quantitative PCR was carried out using PowerUp SYBR Green Master Mix (Thermo Fisher Scientific) in a BioRad CFX96 Real-Time System according to the manufacturer's instructions. The primers sequences used to detect mRNA levels are listed in Table S2. Relative mRNA quantification was calculated with the ΔΔCt method, using *gapdh* as housekeeping gene.

Immunofluorescence Assay

MPCCs were fixed in 4% paraformaldehyde for 20 min at room temperature, washed twice in phosphate-buffered saline (PBS) and stored at 4°C. For CYP3A4 staining, cells were permeabilized with 0.1% TritonX for 5 min at room temperature, washed twice in PBS and blocked with 2% bovine serum albumin (BSA) in PBS for 30 min at room temperature. CYP3A4 monoclonal antibody (Thermo Fisher Scientific Cat# MA5-17064, RRID: AB_2538535) was incubated overnight at 4°C (1:200). For CD81 and ASGPR staining, cells were blocked in 2% BSA and incubated with primary antibodies for 1 hr at room temperature (CD81, Santa Cruz Biotechnology Cat# sc-23962, RRID: AB_627192) or overnight at 4°C (ASGPR, Abcam Cat# ab88042, RRID: AB_10670301). Cells were then washed twice in PBS and incubated with Alexa-conjugated secondary anti-mouse (Molecular Probes Cat# A-31575, RRID: AB_2536185) or anti-rabbit (Molecular Probes Cat# A-11035, RRID: AB_143051) antibodies for 1 hr at room temperature. Cells were washed with PBS, counterstained with Hoechst 33258 (Thermo Fisher Scientific, 1:5 000), and kept in Aquamount (Lerner Laboratories). Images were captured on a Nikon Eclipse T*i* fluorescence microscope and analyzed with ImageJ. Fluorescence intensity in Figure 4D represents corrected total fluorescence (CTF) per island of hepatocytes (10-20 islands scored per condition). CTF = integrated density – (area of selected cell island × mean fluorescence of background readings).

Biochemical Assays

The activity of CYP3A4 in MPCCs was monitored every other day using the cell-based P450-Glo CYP3A4 Assay with luciferin-IPA (Promega) according to the manufacturer's instructions. CYP3A4 activity was also determined by adding testosterone (Sigma-Aldrich, 200 μM) to MPCCs and measuring the formation of its metabolite, 6β-hydroxytestosterone, after 30-min incubation at 37°C. The supernatants were collected, stored at −80°C and later analyzed by liquid chromatography-tandem mass spectrometry at Integrated Analytical Solutions (Berkeley, CA, USA).

Albumin and AAT levels were measured by ELISA. MPCC supernatants were collected and stored at −20°C. Diluted supernatants were incubated with immobilized anti-human albumin or AAT antibodies (Bethyl Cat# A80-129, RRID: AB_67016 and Cat# A80-122, RRID: AB_67027) for 2 hr at room temperature or overnight at 4°C. After washing, plates were incubated with horseradish peroxidase-conjugated anti-human albumin or AAT antibodies (Bethyl Cat# A80-129P, RRID: AB_67023 and Cat# A80-122P, RRID: AB_2285693) for 1 hr at room temperature and developed with tetramethylbenzidine (Thermo Fisher Scientific) per manufacturer's protocols. Albumin and AAT content were determined based on the standard curves included on each plate.

Urea production was measured by colorimetric assay (StanBio Laboratory). Frozen supernatants were mixed with acid and diacetylmonoximine with thiosemicarbazide and incubated at 60°C for 90 min. Acid-catalyzed condensation of urea resulted in a colorimetric product measured by absorbance. Urea content was determined based on standard curves for each plate.

Plasmodium Infection and Quantification

Human hepatocytes were infected with 60 000 *P. falciparum* sporozoites at day 3 post-GalNAc siRNA treatment, in triplicate wells. Cells were washed 3 hr later and maintained in media supplemented with Fungizone (0.25 μg/mL, GIBCO) and penicillin-streptomycin (300 μg/mL, Corning), with daily replacement. MPCCs were fixed in ice-cold methanol for 10 min at 4°C, at day 4 post-infection. Hepatic parasite forms were detected by immunofluorescence assay using *P. falciparum*-specific antibodies (PfHSP70, 1:200 or PfEXP1, 1:100 and PfHSP90, 1:50), as described above. The number of parasites per well was scored manually. The parasite area was measured using NIS-Elements Advanced Research imaging software (Nikon).

QUANTIFICATION AND STATISTICAL ANALYSIS

Statistics were determined with a one-way or two-way ANOVA test with multiple comparisons, using GraphPad Prism software. No formal test was employed to verify whether the data met the assumptions of the statistical approach. Statistical significance was considered for p values below 0.05 (* $p < 0.05$, ** $p < 0.01$, *** $p < 0.001$, **** $p < 0.0001$). Values in bar graphs or scatterplots represent mean ± SEM or SD, and the number of independently performed experiments (n) is indicated in the corresponding figure legend.

Cell Metabolism, Volume *29*

Supplemental Information

Improving Drug Discovery by Nucleic Acid Delivery in Engineered Human Microlivers

Liliana Mancio-Silva, Heather E. Fleming, Alex B. Miller, Stuart Milstein, Abigail Liebow, Patrick Haslett, Laura Sepp-Lorenzino, and Sangeeta N. Bhatia

SUPPLEMENTAL DATA

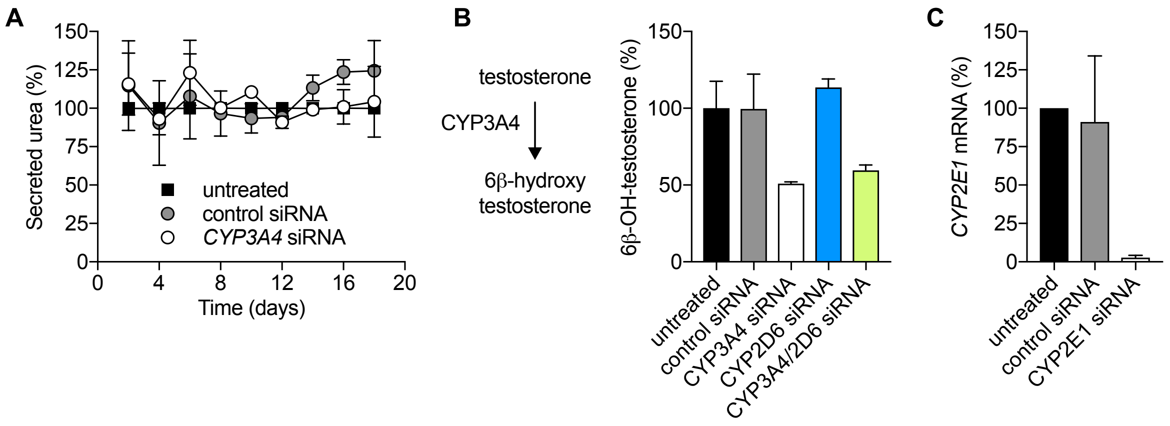

Figure S1. CYP3A4 silencing in MPCCs. **Related to Figures 2 and 3.**
(A) Relative urea levels in the culture supernatant of CYP3A4-silenced MPCCs, as determined by a colorimetric assay. Values are mean ± SEM (n=2). (B) Relative 6β-hydroxytestosterone levels in the supernatant measured by mass spectrometry after incubation with testosterone on day 4 post-transfection (schematic of the reaction shown on the left). 6β-hydroxytestosterone peak area was normalized to untreated cells. Values are mean ± SEM (n=2). (C) *CYP2E1* mRNA levels at day 3 post-transfection. Values (mean ±SEM, n=2) were normalized to untreated MPCCs.

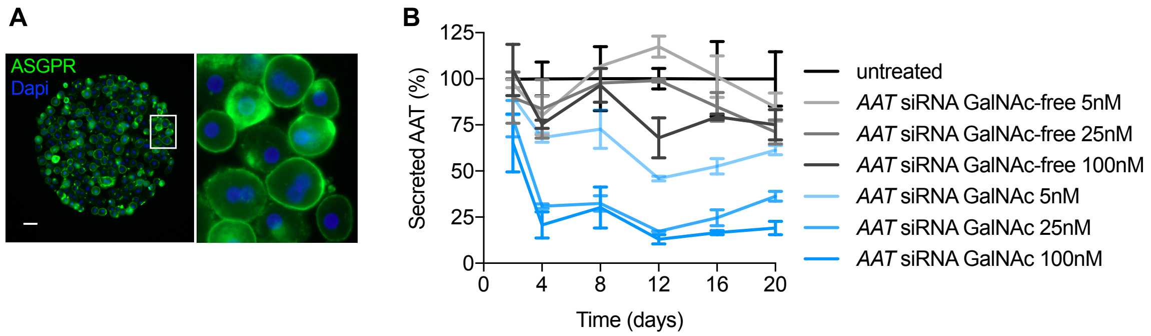

Figure S2. GalNAc-mediated silencing in MPCCs. **Related to Figure 4.**
(A) ASGPR expression in one of the 3 assayed donors at the time of siRNA addition. A representative hepatocyte island is shown, with a zoomed inset on the right. Scale bar, 50 µm. (B) GalNAc- and dose-dependent AAT silencing in free-uptake mode. Values were normalized to untreated cells (mean ± SD). A representative of 3 tested hepatocyte donors is shown.

Table S1. List of GalNAc-conjugated and unconjugated siRNA sequences. **Related to STAR Methods.**

siRNA	Sense (5'-3')	Antisense (3'-5')
CD81	UGCUCUUCGUCUUCAAUUUCA	UGAAAUUGAAGACGAAGAGCAGG
SERPINA1 (AAT)	CACCUGGAAAAUGAACUCACA	UGUGAGUUCAUTUUCCAGGUGCU

Table S2. List of primer sequences. **Related to STAR Methods.**

Primer	Forward	Reverse
CYP3A4	CCGAGTGGATTTCCTTCAGCTG	TGCTCGTGGTTTCATAGCCAGC
CYP2D6	GCAAGAAGTCGCTGGAGCAGTG	CTCACGGCTTTGTCCAAGAGAC
CYP2E1	GAGCACCATCAATCTCTGGACC	CACGGTGATACCGTCCATTGTG
gapdh	GTCTCCTCTGACTTCAACAGCG	ACCACCCTGTTGCTGTAGCCAA
